# Older Adults Aging With HIV: A Growing Population Experiencing Comorbidities and Social Isolation

**DOI:** 10.1093/geroni/igaa057.715

**Published:** 2020-12-16

**Authors:** Carole Treston

**Affiliations:** Association of Nurses in AIDS Care, Phila, Pennsylvania, United States

## Abstract

Significantly more than half of people living with HIV in the United States are over age 50 and at least half of that number are over 70 years old. Advances in antiretroviral treatments continue to extend the lifespan of people with HIV. However, people aging with HIV, particularly those diagnosed earlier in the epidemic, known as “long term survivors” are likely to face a myriad of challenges: clinical, psychosocial, financial, and logistical. Aging with HIV is a complex mix of long-term treatment effects, early onset of general aging, comorbidities and other confounding factors including mental health and psycho-social factors that affect quality of life. Older persons living with HIV have experienced tremendous loss, stigma and discrimination, including within the healthcare system. Now, renewed losses amplified by the emergence of multiple comorbidities including cardiovascular and metabolic disease, HIV associated neurocognitive disease, other neurological disability, diminished bone health and frailty and other conditions can impair quality of life significantly. A review of the common comorbidities experienced by people aging with HIV and the intersection with social isolation, stigma and loss will be presented. Strength based, holistic care that focuses on resilience, and includes advocacy, social networks and care coordination delivered by nurses and nurse practitioners as part of a collaborative inter-professional education program at the Association of Nurses in AIDS Care to address the unique challenges experienced by PLWH will be described.

